# Transparent, Flexible, and Low‐Operating‐Voltage Resistive Switching Memory Based on Al_2_O_3_/IZO Multilayer

**DOI:** 10.1002/gch2.202100118

**Published:** 2022-05-18

**Authors:** Jaemin Park, Daihong Huh, Soomin Son, Wonjoong Kim, Sucheol Ju, Heon Lee

**Affiliations:** ^1^ Department of Materials Science and Engineering Korea University Anam‐ro 145, Seongbuk‐gu Seoul 136‐713 Republic of Korea

**Keywords:** Al _2_O _3_/IZO multilayer, electroforming, resistive switching, transparent memory

## Abstract

In this study, a different number of indium zinc oxide (IZO) interlayers are fabricated into Al_2_O_3_‐based transparent resistive switching memory on a transparent indium tin oxide (ITO)/glass substrate at room temperature. Al_2_O_3_/IZO multilayer transparent memory has a transmittance of at least 65% in the wavelength range of 400–900 nm. In addition, the Al_2_O_3_/IZO multilayer transparent memory can achieve an electroforming voltage that is 35.7% lower than that of ITO/pure‐Al_2_O_3_/IZO transparent memory. The fabricated Al_2_O_3_/IZO multilayer transparent memory exhibits typical bipolar resistive switching behavior, regardless of the number of IZO interlayers. Also, the fabricated Al_2_O_3_/IZO multilayer transparent memory has a low operating voltage within ±1.5 V. In addition, a flexible Al_2_O_3_/IZO multilayer transparent memory is fabricated using the same process on ITO‐coated polyethylene terephthalate. The fabricated flexible transparent memory also maintains the resistive switching characteristics during the bending state.

## Graphical abstract

An Al_2_O_3_/indium zinc oxide (IZO) multilayer transparent resistive switching memory is fabricated and its electrical properties are evaluated. The fabricated Al_2_O_3_/IZO multilayer memory is operated at a low voltage, and the forming voltage of fabricated memory is reduced through the insertion of the IZO layer. In addition, the possibility of a flexible memory is confirmed by fabricating a resistive switching memory on a flexible substrate.

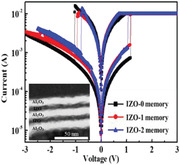

## 1 Introduction

Resistive switching memory has been considered as a candidate for next‐generation memory owing to its simple structure for 3D stacking architecture, high storage density, low power consumption, fast switching speed, and multistate logic potential.^[^ ^1^, ^2^, ^3^, ^4^, ^5^, ^6^, ^7^, ^8^, ^9^ ^]^ Accordingly, many studies related to resistive switching memory have been conducted, and candidate materials for resistive switching memory have been reported such as metal oxides (HfO_2_, SiO_2_, Al_2_O_3_, Ta_2_O_5,_ TiO_2_, etc.),^[^ ^10^, ^11^, ^12^, ^13^, ^14^, ^15^, ^16^ ^]^ organic materials,^[^ ^17^, ^18^, ^19^ ^]^ new‐emerging perovskites,^[^ ^20^, ^21^ ^]^ and 2D materials.^[^ ^22^, ^23^, ^24^ ^]^ Among these, metal oxides have received much attention for use in fabricating resistive switching memory because of their good compatibility with semiconductor manufacturing technologies and low‐cost fabrication. However, in the case of metal oxide‐based resistive switching memory, a relatively high voltage is required to form the nanofilament inside the resistive switching layer in order to operate the resistive switching behavior.^[^ ^14^, ^25^, ^26^ ^]^ Usually, in the electroforming process, a voltage greater than the switching voltage of the resistive switching memory is required. A large electroforming voltage has naturally emerged as a major issue in resistive switching memory and many studies are being conducted to improve the high electroforming voltage issue. To reduce the electroforming voltage, various methods, such as the introduction a heterostructure to facilitate vacancy formation,^[^ ^14^, ^27^, ^28^ ^]^ a thin layer deposition method using atomic layer deposition,^[^ ^29^, ^30^ ^]^ and oxygen vacancy engineering in a metal oxide material,^[^ ^30^, ^31^, ^32^ ^]^ have been studied.

In this study, indium zinc oxide (IZO)/Al_2_O_3_/IZO multilayer/indium tin oxide (ITO) structure of transparent memory was fabricated. An IZO layer was inserted into the Al_2_O_3_ resistive switching layer to facilitate the formation of oxygen vacancy filaments, thereby reducing the electroforming voltage. Furthermore, a transparent and flexible memory was fabricated by applying the Al_2_O_3_/IZO multilayer structure to a transparent electrode. Through electrical characteristic evaluation of the fabricated transparent memory, it was confirmed that the forming voltage was reduced compared to the pure Al_2_O_3_ based transparent memory, and it was confirmed that it had a bipolar switching operation. In addition, through additional evaluation, it was confirmed that a transparent memory with high transmittance in the visible light range was fabricated. The fabricated flexible transparent memory operated well even in a bent state.

## 2 Result and Discussion

**Figure 1:**
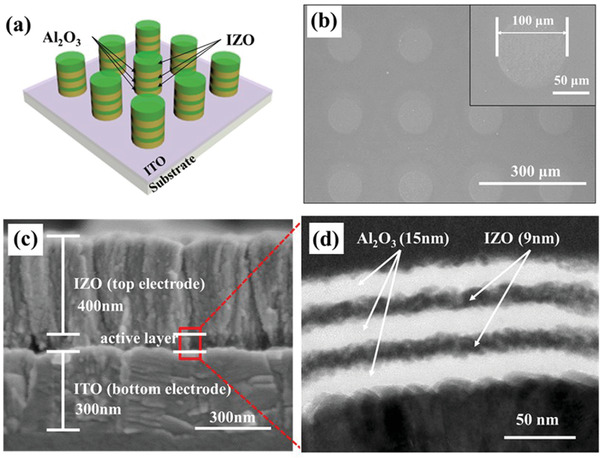
a) Schematic diagram of IZO‐2 memory. b) Top view and c) cross view scanning electron microscope (SEM) images of fabricated IZO‐2 memory. d) Transmission electron microscope (TEM) image of IZO‐2 memory.

**Figure** **1**a shows a schematic diagram of IZO‐2 memory. To fabricate the Al_2_O_3_/IZO multilayer transparent memory, commercial transparent conductive substrate with 300 nm of ITO deposited on a glass substrate (2.5 cm × 2.5 cm) was used. The Al_2_O_3_/IZO multilayers were deposited on ITO glass substrates using a sputter and an e‐beam evaporator at room temperature. A typical disk‐shaped metal shadow mask was used during the deposition process to isolate each memory pattern. The total thickness of the Al_2_O_3_ layer was fixed at 45 nm and an IZO layer with a thickness of 9 nm was inserted into the Al_2_O_3_ layer. To observe the difference according to the number of IZO interlayer, the number of IZO layers was set to 0, 1, and 2, respectively. Figures 1b,c shows the top and cross‐sectional scanning electron microscopy (SEM) images of the IZO‐2 memory. Figure 1b confirms that the diameter of the fabricated IZO‐2 memory is 100 µm. Figure 1c shows that the bottom electrode (ITO) of IZO‐2 memory was deposited at 300 nm and the top electrode (IZO) was also deposited at 300 nm. In addition, it was confirmed that the Al_2_O_3_/IZO multilayer was deposited at 65 nm. Figure 1d shows cross‐sectional transmission electron microscopy (TEM) image of the fabricated IZO‐2 memory. As shown in Figure 1d, the IZO layer was successfully inserted into the Al_2_O_3_ layer. In addition, Figure 1d shows that the thickness of the Al_2_O_3_ layers in the two IZO interlayer transparent memory is 15 nm each, for a total of 45 nm. This shows that the thickness of the IZO interlayer in the Al_2_O_3_ layer is 9 nm. To investigate the components of the IZO‐2 memory in detail, energy‐dispersive X‐ray spectroscopy (EDS) mapping was conducted (Figure S1, Supporting Information).

**Figure 2:**
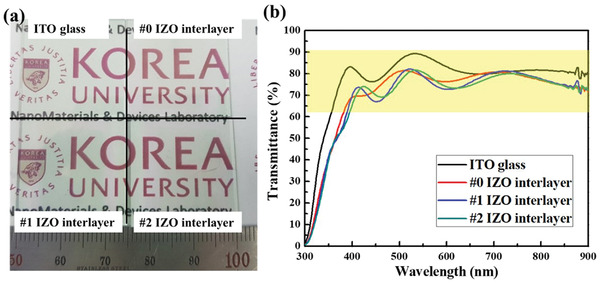
a) Optical image of Al_2_O_3_ based transparent memory with different numbers of IZO interlayers on a Korea University logo. b) Optical transmission spectra of Al_2_O_3_/IZO multilayer transparent resistive switching memory.

**Figure** **2**a presents an optical image of the fabricated transparent memory with different numbers of IZO interlayers. As shown in Figure 2a, it is confirmed that the fabricated devices are sufficiently transparent and the Korea University logo was clearly visible under the fabricated devices. Figure 2b shows that the fabricated Al_2_O_3_ based transparent memory with different numbers of IZO interlayers on ITO glass retained the good optical transmittance of over 65% in the visible region.

The performance of the fabricated Al_2_O_3_‐based transparent memory with different numbers of IZO interlayers was investigated. During the electrical measurements, the bottom electrode (ITO) was grounded and a DC voltage sweep was applied to the top electrode (IZO). Additionally, the compliance current (CC) was set to 10 mA to prevent the transparent memory from breaking down during the electroforming and set processes.

**Figure 3:**
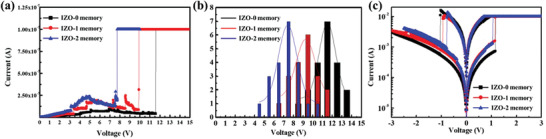
a) Electroforming process of different numbers of IZO interlayers of Al_2_O_3_‐based transparent resistive switching memory. b) Statistical distribution of the forming voltage for Al_2_O_3_‐based transparent resistive switching memory with functional IZO interlayer according to each 20 different cells. c) Electrical switching characteristics of different numbers of IZO interlayers of Al_2_O_3_ based transparent resistive switching memory.

**Figure** **3**a shows the current–voltage (*I*–*V*) characteristic of the electroforming process of the fabricated Al_2_O_3_/IZO memory device with different numbers of IZO interlayers. When the applied current is increased sharply, nanofilaments are formed by the oxygen vacancies created inside the Al_2_O_3_ resistive switching layer, which switches the state of the Al_2_O_3_ resistive switching layer from high‐resistance state (HRS) to low‐resistance state (LRS). In addition, Figure 3a confirms that the electroforming voltage gradually decreases as the number of IZO interlayers increases. Here, the electroforming voltages of IZO‐0, IZO‐1, IZO‐2 Al_2_O_3_ transparent memory are represented by 11.7, 9.8, 7.4 V, respectively Figure 3b shows the statistical distribution of the electroforming voltage of 20 devices corresponding to the number of IZO interlayer with a Gaussian function as a fitting curve. The average electroforming voltages are found to be 11.23, 9.57, 7.21 V for IZO‐0, IZO‐1, IZO‐2 Al_2_O_3_ transparent memory, respectively. Compared to the ITO/pure‐Al_2_O_3_/IZO transparent memory, the average electroforming voltage decreased by 14.7% and 35.7% with one and two IZO interlayers, respectively.

Figure 3c illustrates the semilogarithmic switching *I*–*V* characteristics of the fabricated Al_2_O_3_‐based transparent memory. It is confirmed that typical bipolar resistive switching characteristics exhibited in the different number of IZO interlayer into Al_2_O_3_ layer.^[^ ^31^ ^]^ The reset process was operated by sweeping the voltage in the opposite direction to that of the electroforming process. During the set process, the voltage was applied in the same direction as that of the electroforming process. The voltage was swept from 0 V → −2.5 V → 0 V → 3 V → 0 V. As shown in Figure 3c, it was confirmed that the fabricated transparent memory devices exhibited similar resistive switching behaviors irrespective of the number of IZO interlayers in the Al_2_O_3_ layer. It also shows that the resistive switching characteristics of the fabricated transparent memory devices were operated within ±1.5 V. Resistive switching behavior with a low operating voltage is highly desirable in terms of energy consumption of the memory device.

**Figure 4:**
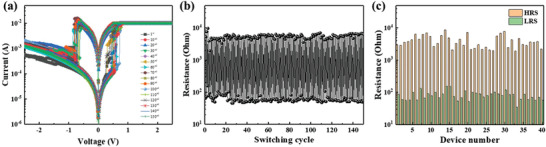
a) Electrical switching characteristics of IZO‐2 memory. The inset shows electroforming process of IZO‐2 memory. b) Endurance characteristics of IZO‐2 memory. c) Resistance of HRS and LRS for 40 different devices.

**Figure** **4**a shows the *I*–*V* curve through a DC sweep to verify the resistive switching hysteresis loop of the IZO‐2 memory. In addition, as shown in Figure 4a, the IZO‐2 memory was operated at a low operating voltage, and stable operation was observed for up to 150 cycles. Figure 4b presents the switching endurance characteristics of IZO‐2 memory up to 150 cycles. The memory resistances at both the HRS and LRS are read at 0.1 V. This shows that the resistance value of the IZO‐2 memory remained constant over the HRS and LRS during the series switching process. From these switching endurance characteristics results, the average *R*_HRS_ was 5.38 × 10^3^, and the average *R*_LRS_ was 6.16 × 10. The average on/off ratio of the fabricated device was greater than 80, which has a sufficient on/off ratio to be applied as a memory device.

The electrical characteristics of 40 different devices were measured to confirm the reliability of the fabricated IZO‐2 memory. Figure 4c shows the resistance values of the HRS and LRS for the 40 different devices. It is confirmed that the IZO‐2 memory has an on/off ratio that is greater than 50 with negligible deviation. The resistance values of Al_2_O_3_ based memory with zero and one IZO interlayer were also measured (Figure S2, Supporting Information).

Resistive switching memory switches resistance states based on the formation and rupture of oxygen vacancy filaments due to a redox reaction in the resistive switching medium when a voltage is applied. The redox reaction can be expressed using the Kröger–Vink notation^[^ ^10^, ^34^, ^35^ ^]^

(1) $$\begin{array}{c} O_{o}\to V_{0}^{\prime \prime }+2e^{-}+\frac{1}{2}O_{2} \end{array}$$

where *V*′′_0_ denotes oxygen vacancy and *O*_o_ denotes oxygen lattice.

**Figure 5:**
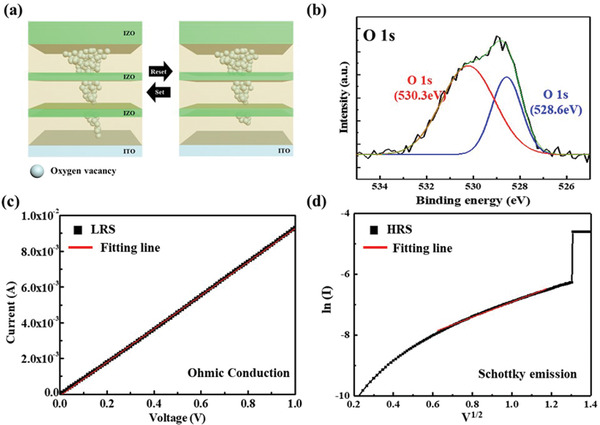
a) Schematic illustration of valence change mechanism for IZO‐2 memory. b) XPS core level spectrum of O 1s in Al_2_O_3_ resistive layer. c) *I*–*V* curve in LRS. d) ln(*I*) – *V* ^1/2^ curve in HRS.

**Figure** **5**a shows the resistive switching process based on the mechanism of conducting filament formation. When a positive voltage is applied, the oxygen ions from the Al_2_O_3_ resistive switching layer migrate to the top electrode and the Schottky barrier between the top electrode and resistive switching layer is reduced. At this time, oxygen vacancies are generated as oxygen ions migrate.^[^ ^34^, ^36^ ^]^ When the generated oxygen vacancy forms a filament and connects between top and bottom electrodes, the resistive switching layer becomes an LRS. Conversely, when a negative voltage is applied, the oxygen ions migrate to the bottom electrode. As the oxygen vacancy migrates, the oxygen vacancy filament that connects the top electrode and the bottom electrode is ruptured, and the resistive switching layer becomes HRS.^[^ ^7^, ^10^, ^37^ ^]^ In the Al_2_O_3_ resistive switching memory with IZO inserting layer, electroforming voltage is reduced. When the filament by oxygen vacancy formed in the first Al_2_O_3_ layer is connected to the IZO layer, electrical stress is applied to the local area connected to the filament. Through this, even when the same voltage is applied, electrical stress is strongly applied to a local area, and the forming voltage of the fabricated Al_2_O_3_/IZO multilayer memory is reduced by facilitating the formation of oxygen vacancy‐based filament. Figure 5b shows the O 1s core‐level spectrum of the X‐ray photoelectron spectroscopy (XPS) of the Al_2_O_3_ resistive switching layer used in the memory. The O 1s spectrum shows an asymmetric distribution, which can be deconvolved into two peaks; this fits the Gaussian distribution with 528.6 and 530.3 eV as peaks. In the XPS data, 528.6 eV is the peak caused by the oxygen confined inside the Al_2_O_3_ lattice. On the other hand, 530.3 eV is the peak created by the oxygen vacancy caused by oxygen escaping from Al_2_O_3_. Through this, it confirmed that the fabricated transparent memory was operating by the filament formation of oxygen vacancy.^[^ ^38^, ^39^ ^]^

Figure 5c,d shows analyses of the conduction mechanism in the IZO‐2 memory in both the LRS and HRS. Figure 5c shows the conduction mechanism in the LRS based on the *I*–*V* plot. Further the linear distribution of the *I*–*V* curve shows ohmic conduction in the LRS. Figure 5d shows the conduction mechanism in the HRS based on the ln(*I*) – *V* ^1/2^ plot. The linear distribution in the ln(*I*) – *V* ^1/2^ plot verifies that HRS follows Schottky conduction.^[^ ^40^, ^41^, ^42^ ^]^ Such conduction mechanisms of LRS and HRS represent the mechanism of oxygen vacancy‐based resistive switching memory.

The fabrication process described in this study is a low‐temperature process and is suitable for fabricating flexible devices. Therefore, Al_2_O_3_/IZO multilayer memories were fabricated on a PET substrate using the same process and their resistive switching characteristics were measured under DC voltage sweeping.

**Figure 6:**
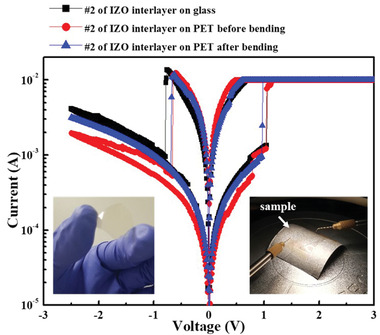
Electrical switching characteristics of flexible IZO‐2 memory. Inset shows photo image of IZO‐2 memory on PET (left) and measurement system of bending device(right) (bending radius: 10 mm).

**Figure** **6**a shows the *I*–*V* characteristics of an IZO‐2 memory fabricated on glass and PET substrates. It was found to exhibit similar behavior, regardless of the substrate used. The *I*–*V* characteristics of IZO‐2 memory fabricated on the PET substrate were compared in the flat and bending states to confirm the flexibility of the electrical characteristics. For the bending measurements, the bending radius was set to 10 mm. The inset (left) shows IZO‐2 memory fabricated on flexible PET, and the inset (right) shows a measurement system during the bending state; they confirm that the resistive switching characteristics before and after bending are almost identical. As shown in Figure 6a, the fabricated flexible transparent memory has a set voltage of 1.1 V and reset voltage of −0.8 V. It is confirmed that it shows similar behavior to the transparent memory on ITO glass mentioned above.

## 3 Conclusion

In this study, we fabricated an Al_2_O_3_ based transparent resistive switching memory with different numbers of IZO interlayers. The fabricated transparent memory has a transmittance of over 65% in the visible region (400–900 nm) and is sufficiently transparent. The fabricated transparent memory exhibited typical bipolar switching characteristics. In addition, it operated at a low voltage between −1.5 and 1.5 V and had a good on/off ratio. In addition, it was confirmed that transparent memory with an IZO interlayer performs similar low‐voltage operation without interrupting the switching behavior compared to the ITO/pure‐Al_2_O_3_/IZO memory. The forming voltage of the fabricated transparent memory decreased as the IZO interlayer increased inside the Al_2_O_3_ layer. Thus, IZO‐2 memory reduces the electroforming voltage by 35.7% compared to the ITO/pure‐Al_2_O_3_/IZO memory. Furthermore, because a high‐temperature process is not required in the fabrication process of transparent memory, transparent memory can be fabricated on a flexible substrate. The switching behavior of the flexible transparent memory was similar to that of the memory fabricated on top of the ITO glass. The IZO‐2 transparent resistive switching memory developed in this study can be applied to transparent and flexible memory devices and is expected to be applicable to wearable and bio‐memory devices in the future.

## 4 Experimental Section

### Fabrication of Al_2_O_3_/IZO Multilayer Transparent Memory

To fabricate the Al_2_O_3_/IZO multilayer transparent memory, a commercial low‐cost ITO‐coated substrate was cleaned by sonication in acetone, ethanol, and DI for 10 min. An Al_2_O_3_/IZO multilayer transparent memory was fabricated using a 100 µm metal shadow mask. The metal shadow mask was attached to the cleaned substrate. Next, Al_2_O_3_ and IZO were alternately deposited. The Al_2_O_3_ layer was deposited using an e‐beam evaporator, and an IZO layer was deposited using a sputter. The total Al_2_O_3_ layer was deposited with a thickness of 30 nm. The deposition rate of the Al_2_O_3_ layer was performed at a uniform 0.3–0.4 Å s^–1^ during the entire process. The IZO interlayer and top electrode were deposited through a sputtering process. An IZO target was used with a sputtering power of 50 W and a working pressure of 3 × 10^–2^ Torr. The thickness of IZO interlayer and top electrode was controlled through sputtering time. Surface treatment was performed through UV–ozone treatment between the Al_2_O_3_ active layer and IZO interlayer. Using the same fabrication method, flexible Al_2_O_3_/IZO multilayer transparent memory was fabricated on an ITO‐coated PET substrate under the same conditions

### Characterization of Al_2_O_3_/IZO Multilayer Transparent Memory

The *I*–*V* characteristic of the device was measured in the voltage sweep using a parameter analyzer (Keithley 4200scs). The bias voltage was applied to the top electrode IZO and the ITO bottom electrode was grounded.

The cross‐section of the Al_2_O_3_/IZO multilayer transparent memory was investigated via field‐emission SEM (Hitachi, S‐4300) and field‐emission TEM (Talos). The samples for the TEM analysis were prepared using a focused ion beam (FET Helios NanoLab 600). The atomic distribution of the samples was measured via X‐ray photo electron spectroscopy (XPS, ULVAC‐PHI/X‐TOOL) with a 100 µm at 1486.6 eV.

## Conflict of Interest

The authors declare no conflict of interest.

## Supporting information

Supporting Information

## Data Availability

The data that support the findings of this study are available in the supplementary material of this article.
