# Supplementary material for: Transparent, Flexible, and Low‐Operating‐Voltage Resistive Switching Memory Based on Al2O3/IZO Multilayer
Source: Glob Chall. 2022 May 18;6(7):2100118. doi: 10.1002/gch2.202100118 (PMC9284630; doi:10.1002/gch2.202100118)
Supplement: Supplementary file 1 — Supporting Information [file GCH2-6-2100118-s001.pdf]

Global Challenges
Open Access

Supporting Information

for *Global Challenges,* DOI: 10.1002/gch2.202100118

Transparent, Flexible, and Low-Operating-Voltage Resistive Switching Memory Based on Al_2O_3/IZO Multilayer

*Jaemin Park, Daihong Huh, Soomin Son, Wonjoong Kim, Sucheol Ju, and Heon Lee**

Supporting Information

Transparent, flexible and low-operating-voltage resistive switching memory based on Al_2O_3/IZO multilayer

Jaemin Park[1], *Daihong Huh*[1], *Soomin Son*[1], *Wonjoong Kim*[1], *Sucheol Ju*[1] *and Heon Lee*[1*]

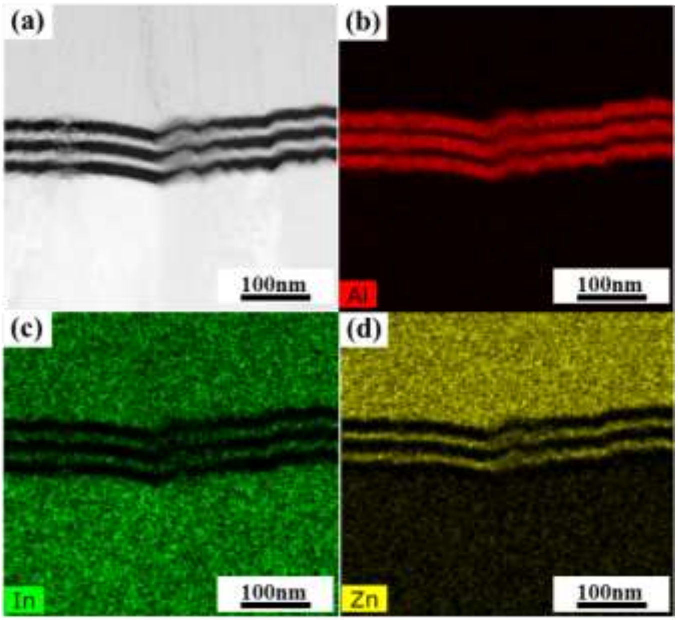

Figure S1. (a) TEM image of the IZO-2 resistive switching memory. (b-d) TEM image of the IZO-2 resistive switching memory corresponding EDX maps of Al, In, Zn.

Figure S1 shows a TEM image of the fabricated IZO-2 resistive switching memory. Through **Figure S1 (b-d)**, it was confirmed that the IZO layer was well inserted into the Al_2O_3 layer.

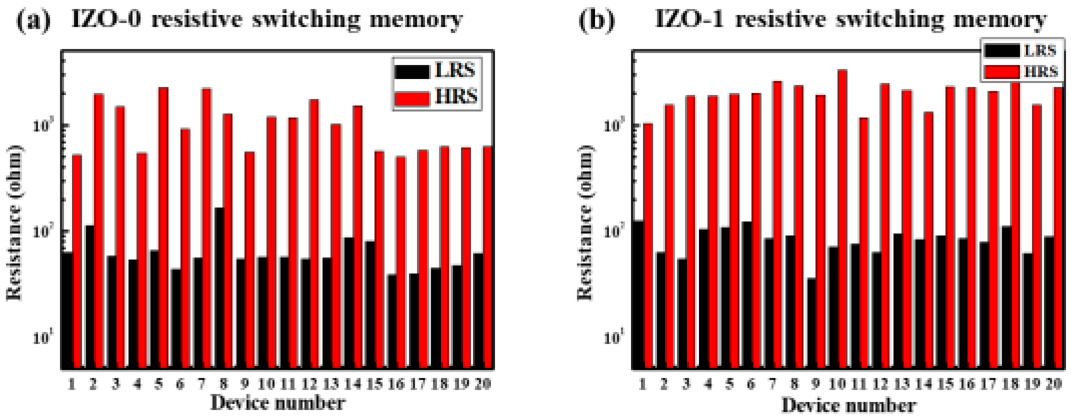

Figure S2. (a) Resistance of HRS and LRS for 20 different devices of IZO-0 resistive switching memory (b) Resistance of HRS and LRS for 20 different devices of IZO-1 resistive switching memory

The electrical characteristics of 20 different devices were measured to confirm the reliability of the fabricated IZO-0, IZO-1 resistive switching memory. **Figure S2** shows the resistance values of the HRS and LRS for the 20 different devices. It is confirmed that the IZO-0, IZO-1 resistive switching memory has an on/off ratio that is greater than 20 with negligible deviation.
